# Impact of the COVID-19 pandemic on Ukrainian mortality, 2020–2021

**DOI:** 10.1371/journal.pone.0285950

**Published:** 2023-05-19

**Authors:** Neil K. Mehta, Ihor Honchar, Olena Doroshenko, Khrystyna Pak, Mariia Daniuk, Pavlo Polikarchuk

**Affiliations:** 1 Department of Epidemiology, School of Public and Population Health, The University of Texas Medical Branch, Galveston, Texas, United States of America; 2 Department of Statistics, Information and Analytical Systems and Demography, Taras Shevchenko National University of Kyiv, Kyiv, Ukraine; 3 Health, Nutrition and Population, World Bank Group, Kyiv, Ukraine; 4 Modern Marketing Communications LTD, Kyiv, Ukraine; Istanbul University-Cerrahpasa Cerrahpasa Faculty of Medicine: Istanbul Universitesi-Cerrahpasa Cerrahpasa Tip Fakultesi, TURKEY

## Abstract

The mortality impact of the COVID-19 pandemic in Ukraine has remained incomplete. We estimated excess deaths associated with the pandemic in Ukraine during 2020 and 2021. Excess deaths may be attributed directly to SARS-CoV-2 infection or indirectly to deaths associated with social and economic upheavals resulting from the pandemic. Data on all deaths registered in government-controlled Ukraine from 2016–2021 (N = 3,657,475) were utilized. Using a model-based approach, we predicted monthly excess deaths in 2020 and 2021. We estimated 47,578 excess deaths in 2020 as a whole (7.71% of all recorded deaths). This figure reflects both positive (higher than expected) excess deaths from June-December and negative (lower than expected) deaths in January and March-May. From June-December 2020, we estimated 59,363 excess deaths (15.75% of all recorded deaths in those months). In 2021, we estimated 150,049 excess deaths (21.01% of all recorded deaths). Positive excess deaths were detected across age groups even groups younger than 40 years. The number of excess deaths exceeded that of deaths with COVID-19 coded on the death certificate by more than two-fold in 2020, but that difference narrowed in 2021. We furthermore provide provisional estimates of the effect of low vaccine coverage on excess deaths in 2021 drawing from European cross-national evidence and provisional estimates of the hypothetical evolution of the pandemic in 2022 to serve as a rough basis for future studies analyzing the joint impacts of the COVID-19 pandemic and the Russian invasion on Ukrainian demography.

## Introduction

Documenting the mortality impact of the COVID-19 pandemic in Ukraine remains incomplete. The number of deaths attributable to the pandemic (i.e., excess deaths) exist only at the national level without specifics on age groups, sex, and regions [1–3]. Moreover, some existing estimates have used the average number of annual deaths in the years leading up to the pandemic as a basis for estimation [1]. While this method provides a useful, albeit rough, picture of the impact of the pandemic as it evolves, it is not the ideal approach and may be inaccurate in situations where there has been considerable demographic change as has been the case in Ukraine [4]. For a more complete assessment, we applied a model-based approach accounting for trends in deaths to estimate the mortality impact of the COVID-19 pandemic as it evolved in Ukraine in 2020 and 2021. We provide estimates at the national level and by region including details on age groups and genders.

Prior to the 2022 Russian invasion of Ukraine on February 24, 2022, COVID-19 vaccine uptake in Ukraine significantly lagged that of other European countries and vaccine uptake after the invasion has been insignificant. We provide provisional estimates of the effect of low vaccine coverage on excess deaths in 2021 drawing from cross-national evidence. We further provide provisional estimates of the hypothetical evolution of the pandemic in 2022 to serve as a rough guide for future studies analyzing the joint impacts of the COVID-19 pandemic and the Russian invasion on Ukrainian demography in 2022 and beyond.

### The evolution of the COVID-19 pandemic in Ukraine, 2020–2021

Ukraine registered its first case of COVID-19 on March 3, 2020. The country implemented its first quarantine on March 12^th^, 2020 [5]. Despite this early intervention, the number of confirmed daily cases grew rapidly to approximately 100 per day at the beginning of April 2020 [6]. Stricter measures were instituted on April 6^th^, 2020, which included the closing of schools, shops, and restrictions on travel [5]. These strict restrictions were in place until May 22^nd^, 2020, when the government eased some restrictions transitioning to a policy of “adaptive quarantine”, which it continued through the Russian invasion. While the growth rate of daily cases slowed after the stricter measures were instituted, daily cases continued to increase reaching a peak of approximately 14,000 per day by the end of November 2020 [6]. By the end of that year, Ukraine was recording approximately 8,100 daily cases. In 2021, Ukraine experienced two significant waves. The first was in early spring where the country recorded 10,630 daily cases in March and 13,301 daily cases in April 2021 [6]. While recorded cases declined during the summer months of 2021, they rose significantly in the fall peaking in November 2021 when approximately 18,280 daily cases were recorded on average [6].

### Regional variation

Like many countries, there was strong regional variation in the spread of the virus. Western regions of the country were the first to record cases due to their proximity to Romania, Poland, and Hungary and high levels of international migration. National restrictions on inter-regional movement during the early part of 2020 likely slowed the spread of the virus within the country protecting eastern and southern regions. The early lifting of these restrictions, however, in May 2020 led to the virus spreading into the eastern and southern parts of the country. As the pandemic evolved, all regions of Ukraine became affected and by 2021 there was more uniformity in per capita cases across regions. Nonetheless, the eastern and southern regions experienced the highest per capita caseloads in 2021 likely attributable to high levels of non-compliance with anti-epidemic rules and low levels of vaccine uptake.

### COVID-19 vaccine implementation

Vaccine uptake in Ukraine as a whole was low compared to other European countries [7, 8]. Vaccines began to be implemented in the population at the end of February 2021, first with the Sinovac COVID-19 vaccine (CoronaVac). As vaccine availability increased throughout the year, AstraZeneca, Pfizer/BioNTech, Moderna, and the Johnson & Johnson vaccines became available. The Sputnik vaccine was not used. By the fall of 2021, all five of vaccines were available. Despite the widespread availability of vaccines by the end of 2021, only 38% of the Ukrainian population were fully vaccinated as of February 22, 2022 [8], compared to 65% for Europe as a whole [7]. There was a preference for the mRNA vaccines and 47% of total doses were Pfizer/BioNTech and 10% were Moderna. Thirty-one percent of doses administered were CoronaVac, most of those were administered during the early part of 2021. Vaccination was mandatory for teachers and civil servants and voluntary for the rest of the population. The low uptake of vaccines among Ukrainians was likely not due to the availability of vaccines, but rather to high levels of hesitancy in the population [9].

### Current study

To date, the impact of the pandemic on Ukrainian mortality remains poorly understood. While estimates of excess deaths attributable to COVID-19 for Ukraine are available online in international databases they do not provide detailed information on age and gender groups [1, 10]. International databases also utilize crude methods for estimating excess deaths, relying on the average number of deaths or death rates in the previous years, which do not account for time trends or differences in trends by age. The assumption from these models is that the number of deaths is stable on a year-to-year basis. While the assumption may be valid for countries whose population size and age structure are relatively stable, it is not valid for countries experiencing stark demographic changes such as fluctuating migration levels, rapid aging, changing mortality, or population decline. Ukraine experienced each of these phenomena in pre-pandemic years.

We estimated monthly excess deaths attributable to the COVID-19 pandemic in Ukraine for 2020 and 2021 and compared these estimates with the number of deaths that had COVID-19 reported on the death certificate. We rely on official vital statistics data that are the most up-to-date available, reflecting all recorded deaths in the population through January 2022 after which records were unavailable. We provide estimates by five-year age groups and gender. In addition, we provide provisional evidence on the mortality impact that the slow vaccination uptake had on the population. We do so by leveraging cross-national data on the relationship between vaccine uptake and excess deaths in a set of European countries. The 2022 Russian invasion of Ukraine undoubtedly changed the course of the pandemic given the large-scale disruptions that it brought to Ukrainian society. We additionally present provisional estimates of the hypothetical impact of the pandemic in 2022, as measured through the lens of excess deaths, if the pandemic continued to evolve as it did in the prior two years.

## Methods

### Data

Our estimates of excess deaths rely on an analysis of aggregated death records. Data from multiple official Ukrainian data sources were utilized. From the Ministry of Justice of Ukraine, we compiled the number of deaths from any cause by age groups (0–4, 5–9, …, 80–84, 85+ years) and sex for each calendar month during 2015–2021. Data on causes-of-death from death certificates were obtained from the State Statistics Service and Ministry of Justice. From these data, we compiled the number monthly deaths with COVID-19 recorded as an underlying cause (ICD-10: U07.1) in 2020 and 2021. All data used were current as of February 2022. Data from regions not controlled by Ukraine (part of Donetsk and Luhansk Regions, full Autonomous Republic of Crimea) during the 2015–2021 period were unavailable. These analyses were entirely based on data that does not include any personal identifying information for any individual and therefore does not constitute human subjects research and ethics consent was not required. All data were available freely to any person until February 24, 2022. Data are available upon request to the State Statistics Service of Ukraine (https://ukrcensus.gov.ua/).

To provide a more complete picture of Ukrainian demography, we separately estimated the age and sex population distribution of the Ukrainian population for 2016–2021. Data for this estimation were obtained from the State Border Guard Service of Ukraine, State Statistics Service of Ukraine, Ministry of Health, and the Public Health Center of Ukraine. Sufficient data for 2015 estimation were unavailable. Official counts of the Ukrainian population by age and sex were available for December 1, 2019 [11]. These counts were estimates of the actual population based on various electronic government registries. The country last administered a full census in 2001. From these December 1, 2019 counts, we projected the population forward into 2020 and 2021 and reconstructed it backward to January 1 2016 using a cohort component method accounting for the flows of natural change (births and deaths) and in- and out-migration [12]. Population estimates were developed by sex and for each single year of age for January 1 of each calendar year during 2016–2021. The population estimates that we produced are pertinent to the Ukrainian controlled areas of the region during the period meaning that they excluded part of Donetsk and Luhansk Regions and the full territory of Autonomous Republic of Crimea and will differ from the World Population Prospects published by the United Nations as those data are pertinent to the entirety of Ukraine.

### Analytical approach

The foundation for calculating the number of excess deaths is the number of deaths that would have occurred in a population in the absence of the pandemic, a counterfactual value. These deaths, which we term basis deaths, are subtracted from actual recorded deaths to obtain the number of excess deaths. Various methods are used to estimate basis deaths. Most common has been to take the mean number of annual deaths in pre-pandemic years and apply that number to the pandemic period. This approach will produce biased estimates when there is an underlying trend in the annual number of deaths attributable to changing population size or changes in the factors that produce mortality. For example, the “average method” will overestimate expected deaths if deaths in the population have been declining over time, resulting in an underestimate of excess deaths. Demographers, rather, often rely on linear or log-linear models to project death data as they often well-capture the smoothness of death rates over time and are transparent in their interpretations [13, 14]. More complex models to predict basis deaths would include covariate predictors (e.g., social and economic conditions, healthcare, weather patterns), however, such data are often not available or are otherwise poorly recorded.

Our approach was to use an ordinary least squares linear regression model in the form $Y_{t}=\beta_{0}+\beta_{1}*Month_{t}$ (eq. 1), which was estimated on the 2015–2019 monthly recorded death data series. In eq. (1), *t* tracks the calendar month with *Month*_*t*_ *=* 1 denoting January 2015 and *Month*_*t*_ = 60 denoting December 2019. *Y*_*t*_ is the number of deaths in month *t* and *β*_0_ is the intercept. *β*_1_ identifies the average change in the absolute number of deaths for a one calendar month increment. It was estimated to be -34.83 reflecting a decrease in deaths over time due to a declining population in the pre-pandemic period. Eq. (1) accounts for smooth (i.e., non-fluctuating) secular changes in number of deaths over the 2015–2019 period. It does not account for normal short-term mortality disturbances that occur over a calendar year due to influenza and other seasonal-based causes of death. To identify these fluctuations, we separately constructed a seasonality index, which is the ratio of number of deaths in a calendar month to the average number of monthly deaths over a calendar year. If deaths in a calendar month were higher than the average monthly deaths over the year, the ratio is greater than 1.00 for that month. The Table in the S1 Appendix shows the index values for each year in 2015–2021.

To check that our model well-predicted actual monthly deaths during 2015–2019, we used eq. (1) to predict death counts in each month and multiplied these predictions by the respective seasonality index to obtain the final monthly death predictions (basis deaths). We calculated the percentage difference between the model-based basis deaths (*B*) and the recorded deaths (*R*)—100x(B-R)/M—for each calendar month during 2015–2019. The 2015–2019 basis and recorded monthly deaths differed by an average of 1.36% with no tendency toward underestimation or overestimation suggesting that the model performed well during the pre-pandemic period. The S2 Appendix shows results from this exercise. The mean standard error of the difference was 2.1%.

Eq. (1) was then used to estimate basis deaths for 2020 and 2021. We set *Month*_*t*_ to its corresponding values in those years (e.g., for January 2020, *Month*_*t*_ = 61 and for December 2022, *Month*_*t*_ = 84) to produce initial monthly predictions of number of deaths. The monthly basis deaths were obtained by multiplying these initial predictions with the corresponding seasonality index value, which was a simple average of the index in 2015–2019 for each month. Monthly number of excess deaths were obtained by subtracting the basis number of deaths from the recorded number of monthly deaths. The percent excess deaths ([number of excess deaths/number of recorded deaths] * 100) was calculated. This value has been termed the P-Score and is often used in assessing COVID-19 excess mortality [15]. Positive values indicated higher-than-expected number (percentage) of deaths and negative values indicated lower-than-expected number (percentage) of deaths. We separately applied this procedure to gender and age groups (0–4 to 80–84 and 85+ years). Those with unknown age were excluded from the age-specific component (less than 0.25% of deaths annually did not have a recorded age). 95% confidence intervals for 2020 and 2021 excess deaths at the national level were calculated from the mean standard error of difference from our validation approach.

Two sets of provisional analyses were conducted. The first was an estimation of the effect of under-vaccination on excess deaths in 2021. Here, we leveraged cross-national variation on vaccine coverage and excess deaths. We implemented a linear regression model in the form $Y_{c}=\beta_{0}+\beta_{1}*Vaccine_{c}$ (eq. 2) for each calendar month separately during July-December of 2021. *Y*_*c*_ is the country-specific percent excess deaths in a given month and *Vaccine*_*c*_ is the country-specific percent of the population fully vaccinated in that month. By stratifying on calendar month, we allowed the magnitude of the association between percent vaccinated and percent excess deaths to vary across time, which is indicated by *β*_1_. Thirty-one countries were used to identify eq. (2) (Austria, Belgium, Bulgaria, Croatia, Cyprus, Czechia, Denmark, Estonia, Finland, France, Germany, Greece, Hungary, Iceland, Ireland, Italy, Latvia, Liechtenstein, Lithuania, Netherlands, Norway, Poland, Portugal, Romania, Slovakia, Slovenia, Spain, Sweden, Switzerland, Ukraine, United Kingdom). We estimated the percent excess deaths that Ukraine would have hypothetically experienced in each month if it had the mean average vaccination level of the other European countries. We did so by calculating the difference between Ukraine’s actual percent vaccinated and the European average for a given month. This difference was multiplied by *β*_1_ to obtain the percentage change in excess deaths for Ukraine. For all countries except Ukraine, monthly percent excess deaths were obtained from Eurostat [16] and percent vaccinated were obtained from Mathieu et al. [7]. For Ukraine, we used our estimates of percent excess deaths and official Ukrainian data on vaccination coverage [8].

Second, we estimated the number of basis deaths and excess deaths in 2022 under a scenario of no Russian invasion. The purpose of these provisional estimates is to serve as a rough guide for future studies analyzing the impacts of the pandemic and the Russian invasion on Ukrainian mortality in 2022. Two sets of death counts were projected for all calendar months in 2022: deaths in the absence of COVID-19 (basis deaths) and deaths that would have occurred if the pandemic continued to evolve (deaths with pandemic, which we term hypothetical recorded deaths). Excess deaths are the difference between these two counts. The basis deaths were projected according to eq. (1) using the same approach we used for 2020 and 2021, but by setting *Month*_*t*_ to its appropriate 2022 value (e.g., *Month*_*t*_ = 85 for January 2022). For January 2022, the last full month that the Ukrainian government recorded deaths, we have actual data on recorded deaths. For the remaining months, we present lower and upper bound estimates for the hypothetical recorded deaths. The lower bound estimate rested on the assumption that the percent excess death that existed for each month of 2021 was the same for the corresponding month in 2022. For example, the percent excess deaths in February 2021 was 8.92%, which we applied to the February 2022 basis deaths using the formula $B\;*\;(\frac{1}{1-E})$ (eq. (3)), where *B* is the basis deaths and *E* is the proportion of excess deaths. The second and upper bound estimate was based on an extrapolation of actual data for January 2022. In that month, the percent excess death was 4.20%, 1.78% higher than in January 2021 (where it was 3.97%). We therefore inflated the 2021 monthly percent excess death series by 1.78% to estimate the 2022 hypothetical recorded deaths. The Russian invasion has resulted in large migration flows, which are not accounted for in our analysis. We therefore additionally present hypothetical recorded deaths in the form of rates (number of deaths per 100,000 population), normalizing by population size, for future analyses conducted when the actual size of the 2022 population is better understood. We estimated the 2022 population using the projection method described above. The January 1, 2022 population was estimated to be 41,167,300.

## Results

Table 1 provides background population characteristics of the government-controlled Ukrainian population for 2016–2019. Between 2016 and 2019 the estimated population declined from approximately 42.59 million persons on January 1, 2016 to approximately 41.42 million on January 1, 2021. Over this period the percentage over aged 65 years increased from 15.89% to 17.41%. Recorded deaths per 100,000 population increased from 1,370 per 100,000 population in 2016 to 1,724 per 100,000 population in 2021. There were 21,284 COVID-19 coded deaths in 2020 and 86,015 COVID-19 coded deaths in 2021.

**Table 1:** Descriptive statistics of the government controlled Ukrainian population, 2016–2021.

	Pre-Pandemic				Pandemic	
Characteristic	2016	2017	2018	2019	2020	2021
Population Size, Number	42,590,879	42,414,905	42,216,766	41,983,564	41,732,779	41,418,717
Males, Number (Percent)	19,717,881 (46.30)	19,644,580 (46.32)	19,558,180 (46.33)	19,455,272 (46.34)	19,343,440 (46.35)	19,195,376 (46.34)
Females, Number (Percent)	22,872,998 (53.70)	22,770,325 (53.68)	22,658,586 (53.67)	22,528,292 (53.66)	22,389,339 (53.65)	22,223,341 (53.66)
Age 65+, Number (Percent)	6,768,862 (15.89)	6,867,534 (16.19)	6,967,270 (16.50)	7,034,551 (16.76)	7,146,499 (17.12)	7,211,190 (17.41)
Recorded Deaths, Number	583,603	574,096	587,624	581,080	616,809	714,263
Recorded Deaths, per 100,000 population	1,370	1,354	1,392	1,384	1,478	1,724
Covid-19 Coded Deaths, Number	-	-	-	-	21,284	86,015
Covid-19 Coded Deaths, per 100,000 population	-	-	-	-	51	208

*Note*: Population size for each calendar year pertains to January 1.

**Fig 1:**
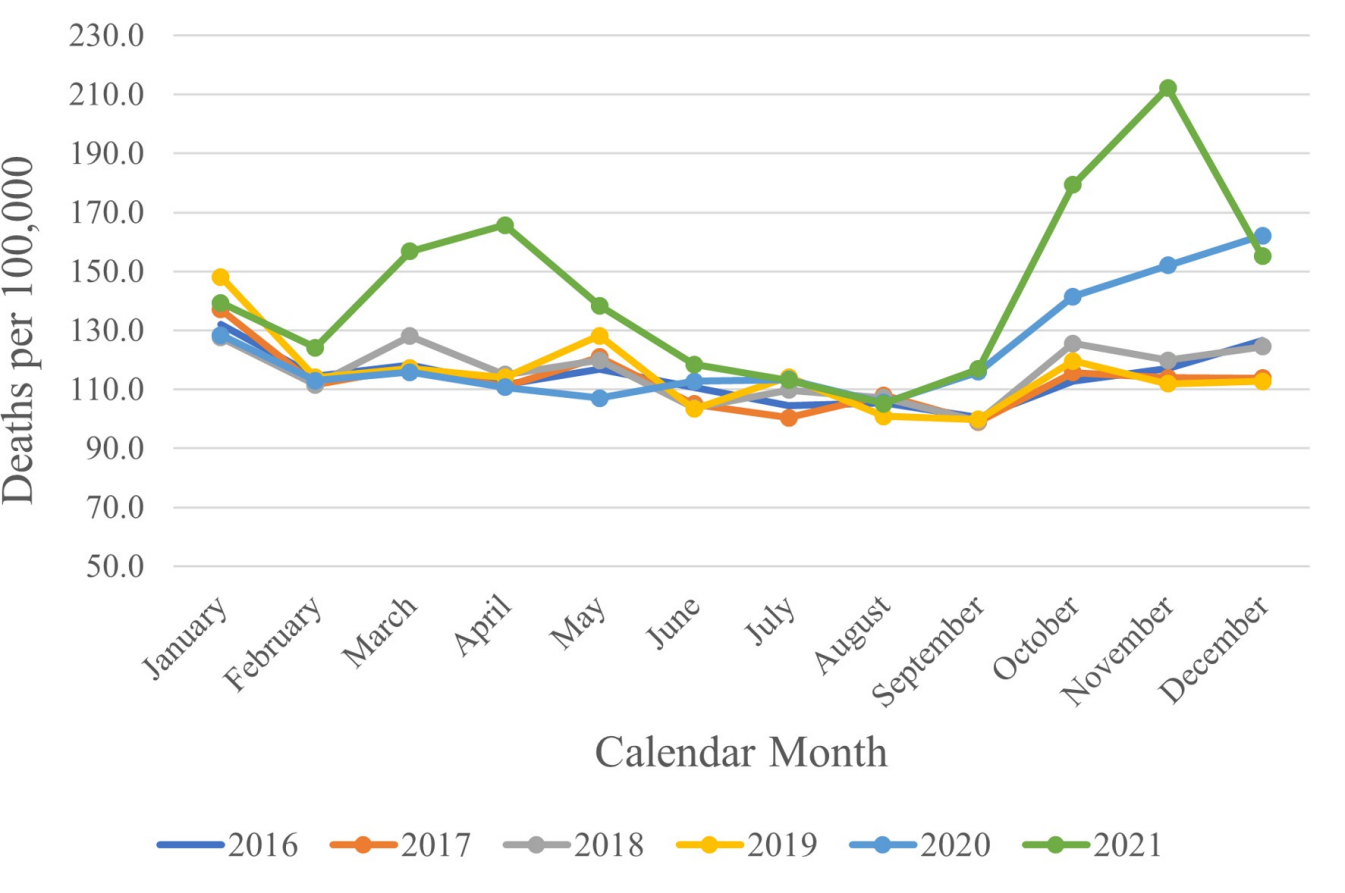
Number of deaths per 100,000 population by calendar month and year in government-controlled Ukraine, all ages and both sexes combined; 2016–2021. Population size as of January 1 of calendar year.

Fig 1 shows number of deaths per 100,000 by calendar month during 2016–2021 highlighting normal seasonal fluctuations in mortality as well as the sizeable increases in mortality during 2020 and 2021 relative to the preceding years.

Table 2 shows the main results for 2020 and 2021. In 2020, Ukraine recorded 1,091,490 COVID-19 cases and 616,809 total deaths. We estimated 569,231 basis deaths for the 2020 calendar year resulting in 47,578 excess deaths (7.71% of all recorded deaths). In four calendar months of 2020 (January, March, April, May), the number of basis deaths exceeded that of recorded deaths resulting in a negative number of excess deaths. Deaths in January of that year were 4.32% lower than expected and those in March and April were 5.23% and 3.64% lower, respectively. Deaths in May 2020 were 11.57% lower than expected. Excess deaths were positive in June 2020 and grew larger in each successive month throughout the remainder of 2020. If we consider only June to December 2020, where the number of excess deaths was positive, 15.75% of all deaths during those months were excess deaths (figure not shown in Table). Males had a higher percentage of excess deaths compared to females (7.81% and 7.62%) in 2020.

**Table 2:** Recorded, basis, and excess deaths in 2020 and 2021 by calendar month; all ages and both sexes combined.

Calendar Month	COVID-19 Cases, number	Recorded Deaths, number	Basis Deaths, number	Excess Deaths, number	Excess Deaths,^1^ percent	COVID-19 Coded Deaths,^2^ number	COVID-19 Coded Deaths,^1^ percent	Ratio of COVID-19 Coded Deaths to Excess Deaths
**2020**								
January	0	53,607	55,921	-2,314	-4.32	0	0.00	-
February	0	47,124	47,218	-94	-0.20	0	0.00	-
March	905	48,321	50,847	-2,526	-5.23	9	0.02	-
April	10,706	46,221	47,902	-1,681	-3.64	243	0.53	-
May	13,193	44,675	49,845	-5,170	-11.57	411	0.92	-
June	22,404	47,042	43,814	3,228	6.86	449	0.95	0.14
July	27,488	47,297	44,711	2,586	5.47	525	1.11	0.20
August	56,052	44,008	43,055	953	2.17	928	2.11	0.97
September	103,757	48,371	41,260	7,111	14.70	1,735	3.59	0.24
October	225,122	59,045	48,435	10,610	17.97	4,050	6.86	0.38
November	346,672	63,436	47,171	16,265	25.64	6,184	9.75	0.38
December	285,191	67,662	49,052	18,610	27.50	6,750	9.98	0.36
**Total**	**1,091,490**	**616,809**	**569,231**	**47,578**	**7.71**	**21,284**	**3.45**	**0.45**
**Total-Females**	**n/a**	**309,101**	**285,561**	**23,540**	**7.62**	**11,505**	**3.72**	**0.49**
**Total-Males**	**n/a**	**307,708**	**283,670**	**24,038**	**7.81**	**9,779**	**3.18**	**0.41**
**2021**								
January	174,549	57,719	55,430	2,289	3.97	3,922	6.79	1.71
February	133,982	51,389	46,803	4,586	8.92	3,014	5.87	0.66
March	329,501	64,937	50,400	14,537	22.39	8,104	12.48	0.56
April	399,041	68,617	47,481	21,136	30.80	12,545	18.28	0.59
May	136,829	57,293	49,406	7,887	13.77	6,327	11.04	0.80
June	39,202	49,075	43,428	5,647	11.51	1,762	3.59	0.31
July	33,308	46,849	44,317	2,532	5.40	599	1.28	0.24
August	51,155	43,536	42,675	861	1.98	804	1.85	0.93
September	148,737	48,422	40,895	7,527	15.54	2,937	6.07	0.39
October	524,809	74,279	48,007	26,272	35.37	15,068	20.29	0.57
November	548,514	87,883	46,754	41,129	46.80	21,867	24.88	0.53
December	240,604	64,264	48,618	15,646	24.35	9,066	14.11	0.58
**Total**	**2,760,231**	**714,263**	**564,214**	**150,049**	**21.01**	**86,015**	**12.04**	**0.57**
**Total-Females**	**n/a**	**370,147**	**277,579**	**92,568**	**25.01**	**47,714**	**12.89**	**0.52**
**Total-Males**	**n/a**	**344,116**	**286,635**	**57,481**	**16.70**	**38,301**	**11.13**	**0.67**

*Note*: Excess deaths are recorded deaths minus basis deaths. Basis deaths were predicted from eq. (1) and the seasonal index. Number of COVID-19 cases were from Public Health Center of Ukraine (https://www.phc.org.ua/). COVID-19 cases were not available by sex.

^1^ As a percent of recorded deaths

^2^ COVID-19 coded deaths are from death certificate data with COVID-19 (ICD-10: U07.1) listed as the underlying cause

n/a: Not available

In 2021, number of excess deaths were positive in every calendar month and were considerably higher than the corresponding months in 2020 (Table 2). In total, there were an estimated 150,049 excess deaths in 2021 representing 21.01% of all recorded deaths. As in 2020, there was an ebb in excess deaths during the summer months followed by an increase in the fall. November 2021 had the highest number and percentage of excess deaths—in that month 46.80% (41,129) of all recorded deaths were excess deaths. Females experienced higher percent excess deaths compared to males during the calendar year (25.01% vs 16.70%).

Table 2 also shows the monthly number of deaths coded as COVID-19 on the death certificate and the monthly ratio of the number of COVID-19 coded deaths to number of excess deaths (restricted to months in which excess deaths were positive). This ratio was 0.45 in 2020 and 0.57 in 2021. The ratio generally increased over time.

S3 Appendix shows 95% Confidence Intervals for basis deaths in 2020 and 2021. S4 Appendix shows Ukrainian monthly percent excess deaths in the context of its nearest neighbors (Poland, Romania, Hungary, Slovakia) and averages across 30 European countries.

**Fig 2:**
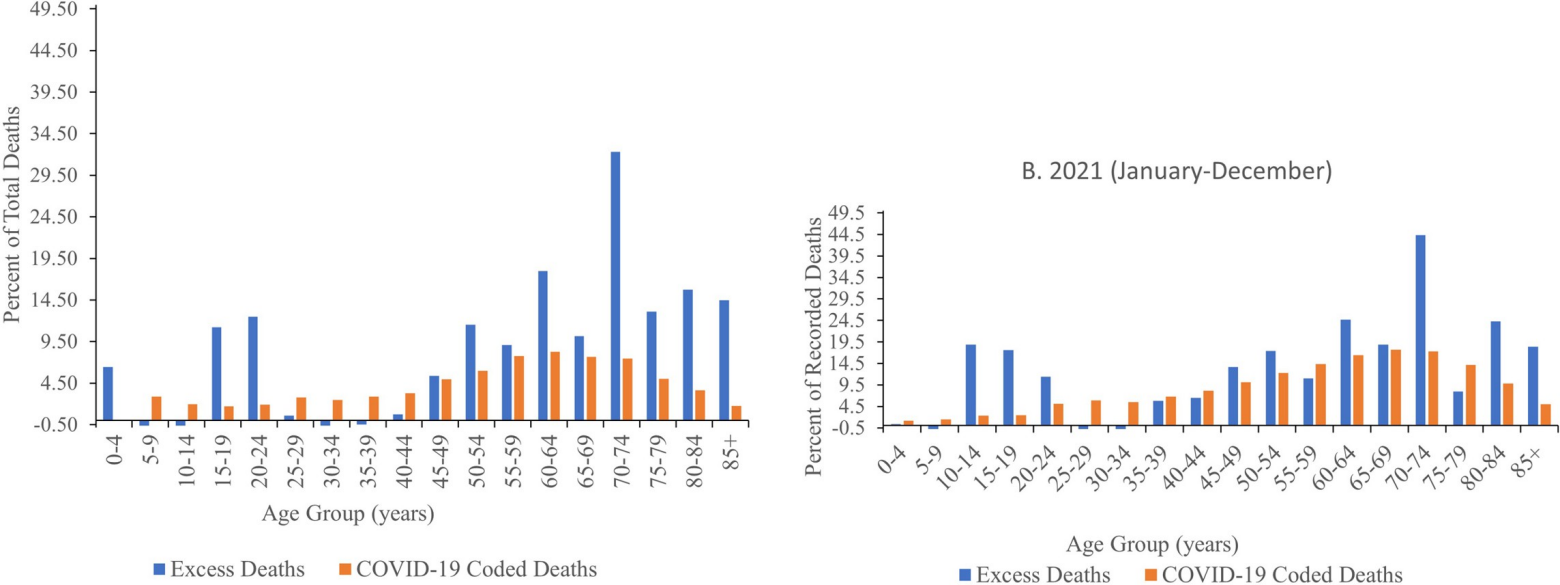
Percent excess deaths and COVID-19 coded deaths by age group in (a) 2020 and (b) 2021. Estimates for 2020 include only months that had positive excess deaths across all ages (June-December).

**Fig 3:**
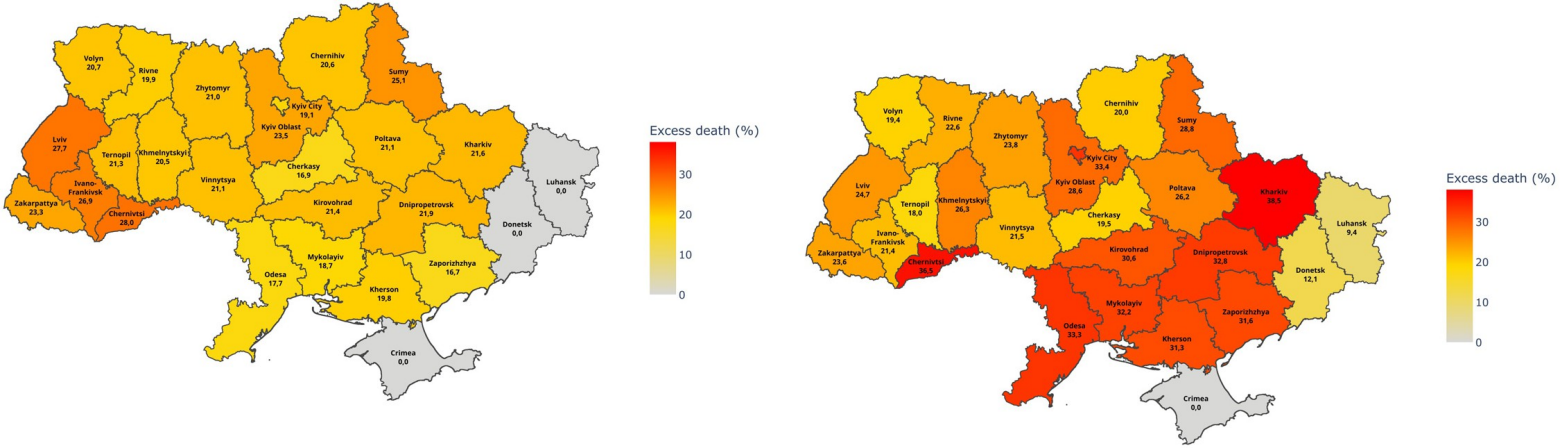
Heat maps of percent excess deaths by region and calendar year in (a) 2020 and (b) 2021. Data for Luhansk and Donetsk reflect only those parts of these regions that were controlled by the Ukrainian government during these years. Percent excess deaths were from authors’ analysis of mortality data from the State Statistics Service of Ukraine. Basemaps obtained from Cartography Vectors (https://cartographyvectors.com/map/1530-ukraine-with-regions). Python version 3.11.1 was used to construct heat map shading.

Age-specific percent excess deaths and percent COVID-19 coded deaths are presented in Fig 2A (2020) and 2b (2021). S5 Appendix (2020) and S6 Appendix (2021) shows the underlying numbers, including the 95% confidence intervals for the predicted basis deaths. Data for 2020 is limited to June-December where the all-age number of excess deaths were positive. It is notable that positive excess deaths were not limited to those over age 50 years in 2020. In June-December 2020, all age groups over ages 35–39 years experienced positive percent excess deaths with the oldest ages experiencing the largest percentages. The absolute number of recorded deaths in the younger age groups was relatively small so although these groups experienced positive excess deaths the absolute number of excess deaths was also small. For example, among those aged 20–24 there were 938 deaths recorded from June to December 2020 and 821 basis deaths (95% CI: 688, 954) resulting in 12.47% excess deaths. Nonetheless, uncertainty in the basis death prediction indicates that the excess deaths for all age groups under age 50 years is not significantly different than zero at the p = .05 threshold (S5 Appendix). Those ages 70–74 years experienced the highest percentage—32.32% in June-December 2020. The age pattern in 2021 (Fig 3B) was similar to that of June-December 2020, however, excess deaths at each age were generally higher. Similar to June-December 2020, there were positive excess deaths at ages younger than 50 years (namely in the 10–14, 15–19, 20–24 age groups), however, these estimates were also not significantly different than zero at the p = .05 level (S6 Appendix).

Fig 3A (2020) and 3b (2021) show region-specific percent excess deaths using heat maps. In 2020, the western regions of the country and those bordering Poland, Slovakia, Hungary, and Romania experienced the highest percent excess deaths. Other regions that experienced relatively high levels in 2020 were the Kyiv Oblast, which contains the Kyiv metropolitan area and the northern region of Sumy, which borders Russia. The lowest affected regions tended to be in the southeast of the country. While all regions experienced higher percent excess death in 2021 compared to 2020, the most affected regions in 2021 were the southeastern and eastern regions. The metropolitan Kyiv Oblast continued to experience relatively high percentages in 2021 with 29% of all recorded deaths being excess deaths. The underlying values to produce the heat maps are provided in S7 Appendix (2020) and S8 Appendix (2021), which also show the monthly percent excess deaths.

Table 3 provides provisional estimates of the effect of low vaccine coverage in Ukraine relative to other European countries for select months in 2021. In September through December of 2021 the cross-country association of vaccine coverage with percent excess deaths was negative implying that an increase in vaccination coverage resulted in a reduction of the percentage of excess deaths. The magnitude of this association was strongest in October and November where caseloads were high across Europe. Based on the cross-national pattern, if Ukraine would have achieved mean European vaccination levels it would have reduced its excess death burden in 2021 by 15,712 (October), 27,381 (November), and 6,093 (December).

**Table 3:** Regression results, percent vaccinated, percent excess deaths and estimates of lives saved if Ukrainian vaccination levels reached that of the European average, July 2021-November 2021.

Calendar Month, 2021	Beta	SE	Intercept	European (Percent vaccinated, mean)	Ukraine (Percent vaccinated)	Ukraine, Percent Excess Deaths	Ukraine, Percent Excess Deaths if European Vaccination Mean	Number of Lives Saved if European Vaccination Mean
July	0.21	0.19	6.79	34.32	2.02	5.71	-	-
August	0.21	0.21	10.24	45.79	4.68	2.01	-	-
September	-0.43**	0.14	43.44	53.79	9.03	18.40	14.87	1,445
October	-1.34***	0.21	12.67	58.01	13.23	54.73	22.00	15,712
November	-1.50***	0.18	11.30	60.61	17.34	87.97	29,40	27,381
December	-1.06***	0.16	15.34	62.99	26.31	32.18	19.65	6,093

*Note*: Beta is *β*_1_ from eq. 2 indicating the magnitude of the association between percent of the population vaccinated and percent excess death in each calendar month. SE is the standard error of Beta.

** p < .001;

*** p < .0001

Table 4 shows provisional estimates for 2022. Basis deaths are lower in 2022 compared to 2021 due to long-run declines in population size. We estimated that the country would have recorded 707,953 (lower bound) to 711,889 (higher bound) deaths in 2022 with 148,754 (lower bound) and 152,690 (higher bound) excess deaths. These figures translate into an average 143.31 to 144.10 monthly deaths per 100,000 population in 2022.

**Table 4:** Provisional estimates of basis deaths, hypothetical recorded deaths, and excess deaths for 2022 under the assumption of no Russian invasion.

Calendar Month	(A) Percent Excess Death, 2021	(B) Basis Deaths, 2022	(C) Hypothetical Recorded Deaths, Lower Bound, 2022	(D) Hypothetical Recorded Deaths, Upper Bound, 2022	(E) Hypothetical Recorded Death Rate, Lower Bound,^1^ 2022	(F) Hypothetical Recorded Death Rate, Upper Bound,^1^ 2022	(G) Excess Deaths, Lower Bound, 2022	(H) Excess Deaths, Upper Bound, 2022
January	3.97	54,939	57,248	-	139.06	-	2,309	-
February	8.92	46,389	50,934	51,023	123.73	123.94	4,545	4,634
March	22.39	49,953	64,361	64,693	156.34	157.15	14,408	14,740
April	30.80	47,060	68,009	68,552	165.20	166.52	20,949	21,492
May	13.77	48,967	56,784	56,946	137.93	138.33	7,817	7,979
June	11.51	43,042	48,639	48,752	118.15	118.42	5,597	5,710
July	5.40	43,923	46,432	46,480	112.79	112.90	2,509	2,557
August	1.98	42,295	43,148	43,164	104.81	104.85	853	869
September	15.54	40,531	47,991	48,149	116.58	116.96	7,460	7,618
October	35.37	47,579	73,617	74,341	178.82	180.58	26,038	26,762
November	46.80	46,337	87,099	88,485	211.57	214.94	40,762	42,148
December	24.35	48,184	63,690	64,057	154.71	155.60	15,506	15,873
**Total**	**21.01**	**559,199**	**707,953**	**711,889**	**143.31**	**144.10**	**148,754**	**152,690**

*Note*: Basis deaths are estimated from eq. 1 and represent the expected number of deaths that would occur in the absence of the COVID-19 pandemic and the Russian invasion. Hypothetical recorded deaths are predictions of what would occur if the COVID-19 pandemic had evolved without the Russian invasion. Deaths in January 2022 were actual recorded deaths from vital statistics.

^1^ Monthly deaths per 100,000 population. January 1 2022 population size was estimated to be 41,167,300. Total is an average of the 12 months.

## Discussion

Using a model-based approach, we estimated monthly excess deaths in Ukraine for 2020 and 2021. We additionally provided provisional estimates of the impact of poor vaccination coverage in 2021 and, for 2022, basis and hypothetical recorded deaths for future studies. For 2020, the model resulted in an estimate of 47,578 excess deaths and 59,363 excess deaths if we consider only June to December of that year when excess deaths were positive. The 59,363 figure represents 15.75% of all recorded deaths that occurred during June-December 2020. For 2021, all calendar months had positive excess deaths with a total of 150,049 excess deaths or 21.01% of all recorded deaths in the calendar year.

These results need to be interpreted in the context of the strengths and limitations of the excess death metric and our modeling approach. The main strength of the metric is that it does not rely on the capacity of a country to detect all COVID-19 cases and code deaths resulting from SARS-CoV-2 infection on the death certificate accordingly. The metric also provides a yardstick to measure the full death toll of the pandemic including deaths directly attributable to the virus and deaths attributable to the broader social and economic shocks that nations underwent. By incorporating recent trends in our model, rather than the “average” approach that is commonly used, we leveraged trends in mortality trends. The “average” approach using 2015–2019 data would have resulted in an estimate of 26,200 excess deaths for 2020, considerably smaller than our estimate of 38,095 deaths. More complex modelling approaches that incorporate additional variables (e.g., weather, health system capacity, etc.) may produce different estimates of excess deaths, but can suffer from significant measurement error, as can models that use more complex functions to extrapolate historical death trends. A main limitation of the excess death metric is that it does not separate deaths directly attributable to SARS-CoV-2 infection from deaths that may be associated with social and economic upheavals created by the pandemic.

The provisional estimates of the effect of under-vaccination and deaths in 2022 must be interpreted with caution. The under-vaccination effects were based on a cross-sectional model of the relation between vaccination and excess deaths and does not account for lags in this relationship. It should not be considered a causal effect. Moreover, we relied on Eurostat data for these estimates, which relied on the “average method” to compute the percent of excess deaths, which as we have described above should not be considered the gold standard to identify COVID-19 excess mortality. The 2022 death estimates were based on the hypothetical evolution of the pandemic given patterns existing in 2021, which do not account for changes in virus strains and population immunity and behavior independent of vaccination. The upper bound estimate was based on an extrapolation from a single month, January 2022, and thus is subject to stochastic uncertainty.

There has been limited information on excess deaths in Ukraine published in the academic literature. Prior estimates have only been at the national level and have ranged from approximately 178,000 to 198,000 for 2020–2021 [2, 3, 17]. Our combined estimate was 197,627. We used the most up-to-date official death statistics available prior to the Russian invasion and our model predicted yearly and seasonal deaths in 2015–2019 for Ukraine with a high degree of accuracy. Thus, the estimated basis deaths for 2020 and 2021 from which excess deaths are calculated from are likely of high quality.

Ukraine’s aggressive action in terms of limiting social interactions in March of 2020 limited deaths during this early period. Unlike other European countries, Ukraine did not experience an early death surge. The country recorded its first confirmed COVID-19 coded deaths in March (9 coded deaths). Although 252 COVID-19 coded deaths were recorded in March and April 2020 combined, our estimates indicated that Ukraine experienced approximately 4,207 *less* deaths than would be expected in these two months. An additional 5,170 negative excess deaths were estimated for May 2020. We can speculate that these “negative” estimates are attributable to a combination of lower mortality from influenza and reduced deaths from accidents and injuries resulting from restriction of movement. International evidence points to 2020 as having below average influenza activity as social distancing restrictions and severe limitations on international travel in response to the COVID-19 pandemic reduced global influenza spread [18]. Aside from reduced influenza, reduced deaths from traffic and other accidents and injuries associated with public interactions may also have been an important factor in reducing excess mortality [19]. Future studies examining causes of death will help identify the sources of the negative excess deaths.

Ukraine began experiencing sustained positive excess deaths in June 2020, soon after the easing of restrictions at the end of May. Similar to the growth in recorded COVID-19 cases, excess deaths continued to increase at a steady pace reaching a peak in November 2020. In that month, excess mortality was estimated to be 25.64%, a level comparable to the European Union as a whole in April 2020 [1], but lower than peak estimates observed in the hardest hit countries. For example, the United Kingdom reached a peak excess mortality of 108% in the middle of April 2020 [10].

National-level excess deaths were low in the beginning of 2021, but the country experienced two significant waves—one in early spring and then one in early fall. Excess deaths were higher in each month of 2021 compared to the corresponding month in 2020 suggesting that although the virus displayed a wave-like epidemic in both years, there was sufficient virus circulating throughout 2021 to produce positive excess deaths in each calendar month. As vaccines became available, we would expect excess deaths to have declined. However, as noted, Ukrainian vaccination levels remained well under the European average. As of January 3 2022, approximately 33% [8] of the population was fully vaccinated compared to the European average of 63% at the end of 2021 [7]. We estimated that the low vaccination level of Ukraine vis-à-vis the European average resulted in 50,631 extra deaths in 2021.

There was a stark shift in the regional pattern of excess deaths between 2020 and 2021. In 2020, the western part of the country and Kyiv Oblast experienced the highest percent excess deaths likely because these areas had high levels of migration to and from other countries. In 2021, the highest percentage of excess deaths were estimated to be in the eastern and southern regions. We speculate that these regions experienced the highest mortality because of stronger resistance to COVID-19 prevention protocols and poorer medical services compared to other parts of the country. Note that estimates for the Luhansk and Donetsk regions reflect only data for portions of these regions that the Ukrainian government controlled in 2020 and 2021. We estimated 0 excess deaths for these regions in 2020 (and 9% and 12% in Luhansk and Donetsk, respectively, in 2021). This estimate may be reflective of low virus activity during the year as both regions experienced conflict and there was little in- and out-migration. However, data quality from these regions was very poor in 2020. Data quality was better in 2021.

We found that COVID-19 coded deaths on the death certificate were approximately 45% (ratio of 0.45) of the estimates of excess deaths for June to December of 2020. This finding is in line with studies from other countries that show that estimates of excess deaths often far exceed that of COVID-19 coded deaths [20, 21]. The main conclusion is that reliance on COVID-19 coded deaths will substantially under-estimate the total mortality impact of the pandemic. One likely source for the discrepancy between the two figures is that not all cases and deaths attributable to COVID-19 infection are detected because of insufficient testing. Another source is that excess deaths capture deaths directly attributable to infection as well as those indirectly caused by the social and economic upheavals of the pandemic. Nonetheless, the ratio improved to 0.57 in 2021 suggesting that the health system became more apt at recording COVID-19 deaths over time.

Our estimates also highlight that excess mortality was not limited to older age groups, although older-aged groups experienced the highest absolute increases in mortality. We estimated that those in age groups 0–4, 15–19, and 20–24 experienced positive excess deaths, however, our results cannot be conclusive given the uncertainty in our modelling approach and the positive excess deaths observed at younger ages were not significantly different than zero at the p = .05 threshold. While younger-aged groups may be at low risk of death from the virus itself, they may have been adversely affected by quarantine restrictions and effects on healthcare. For example, higher infant mortality may be related to poorer pre- and peri-natal care due to overburdened health systems or fear of accessing healthcare. For those in their teenage and early adulthood years, social isolation may have increased suicide risks. Domestic violence may have also increased along with other forms of violence. It is imperative that future studies carefully examine potentially positive excess mortality in these younger age groups.

Russia’s full-scale invasion of Ukraine caused a massive disruption to Ukrainian society. While the outcome of the invasion and its demographic consequences may not be known soon, we provided a set of provisional estimates of excess deaths that may have occurred in the absence of the invasion to aid future studies in identifying the full demographic impact of the invasion. As indicated, these are provisional estimates with many assumptions. We provide estimates in the form of death rates (deaths per 100,000 persons) as the 2022 population size of Ukraine is unknown but will likely be much smaller due to high levels of out-migration to Europe. It is estimated that about 4 million Ukrainians have left the country since the beginning of the invasion, and an additional estimated 7 million persons are internally displaced. Many individuals have migrated from the western and southeastern portions of the country, areas that have had poor vaccination coverage creating risks for generating new epidemics in other European countries. A complete demographic accounting of the Ukrainian population will require a coordination across European countries in terms of counting refugees as well as a full census within the country. International organizations have an important role to play in coordinating these efforts.

## Supporting information

S1 Appendix Seasonality index by calendar month in government-controlled Ukraine, all ages and both sexes combined; 2015–2021. (DOCX)

S2 Appendix Model-based predicted and actual deaths by calendar month, 2015–2022. (DOCX)

S3 Appendix 95% Confidence intervals for the monthly basis death estimates shown in Table 2. (DOCX)

S4 Appendix Percent excess deaths in Ukraine and other European countries, 2020. (DOCX)

S5 Appendix Recorded, basis, and excess deaths in 2020 by age group for June-December 2020; both sexes combined. (DOCX)

S6 Appendix Recorded, basis, and excess deaths in 2021 by age group; both sexes combined. (DOCX)

S7 Appendix Percent excess deaths by region and month, 2020. (DOCX)

S8 Appendix Percent excess deaths by region and month, 2021. (DOCX)
