# Supplementary material for: Impact of the COVID-19 pandemic on Ukrainian mortality, 2020–2021
Source: PLoS One. 2023 May 19;18(5):e0285950. doi: 10.1371/journal.pone.0285950 (PMC10198475; doi:10.1371/journal.pone.0285950)
Supplement: S1 Appendix — (DOCX) [file pone.0285950.s001.docx]

**S1 Appendix**. Seasonality index by calendar month in government-controlled Ukraine, all ages and both sexes combined; 2015-2021

**
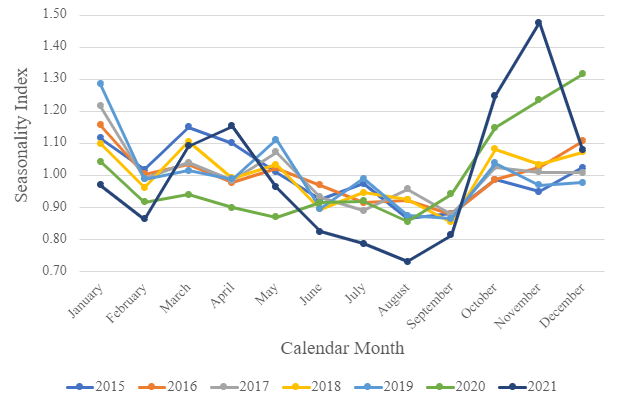
**

Note: Seasonality index is the ratio of number of deaths in a calendar month to the average number of monthly deaths over a calendar year.
