# Supplementary material for: Impact of the COVID-19 pandemic on Ukrainian mortality, 2020–2021
Source: PLoS One. 2023 May 19;18(5):e0285950. doi: 10.1371/journal.pone.0285950 (PMC10198475; doi:10.1371/journal.pone.0285950)
Supplement: S2 Appendix — (DOCX) [file pone.0285950.s002.docx]

**S2 Appendix.** Model-based predicted and actual deaths by calendar month, 2015-2022

*Note*: Both sexes and all ages combined. Blue line shows actual recorded deaths. Predicted deaths (orange line) are obtained from eq. (1) and the monthly mortality fluctuation index.
