# Supplementary material for: Impact of the COVID-19 pandemic on Ukrainian mortality, 2020–2021
Source: PLoS One. 2023 May 19;18(5):e0285950. doi: 10.1371/journal.pone.0285950 (PMC10198475; doi:10.1371/journal.pone.0285950)
Supplement: S3 Appendix — (DOCX) [file pone.0285950.s003.docx]

**S3 Appendix.** 95% Confidence intervals for the monthly basis death estimates shown in Table 2.

| Calendar  Month | Basis Deaths, number  From Table 2 | Lower Bound | Upper  Bound |
| --- | --- | --- | --- |
| **2020** |  |  |  |
| January | 55,921 | 51,607 | 60,234 |
| February | 47,218 | 42,898 | 51,538 |
| March | 50,847 | 46,519 | 55,174 |
| April | 47,902 | 43,567 | 52,237 |
| May | 49,845 | 45,502 | 54,187 |
| June | 43,814 | 39,464 | 48,164 |
| July | 44,711 | 40,353 | 49,069 |
| August | 43,055 | 38,688 | 47,421 |
| September | 41,260 | 36,885 | 45,635 |
| October | 48,435 | 44,051 | 52,818 |
| November | 47,171 | 42,779 | 51,564 |
| December | 49,052 | 44,651 | 53,453 |
| Total—all months | **569,230** | **516,964** | **621,495** |
|  |  |  |  |
| **2021** |  |  |  |
| January | 55,430 | 51,019 | 59,840 |
| February | 46,803 | 42,383 | 51,223 |
| March | 50,400 | 45,970 | 54,830 |
| April | 47,481 | 43,041 | 51,920 |
| May | 49,406 | 44,956 | 53,855 |
| June | 43,428 | 38,968 | 47,888 |
| July | 44,317 | 39,847 | 48,787 |
| August | 42,675 | 38,194 | 47,155 |
| September | 40,895 | 36,404 | 45,387 |
| October | 48,007 | 43,504 | 52,509 |
| November | 46,754 | 42,241 | 51,268 |
| December | 48,618 | 44,093 | 53,143 |
| Total—all months | **564,214** | **510,622** | **617,806** |
