# Supplementary material for: Impact of the COVID-19 pandemic on Ukrainian mortality, 2020–2021
Source: PLoS One. 2023 May 19;18(5):e0285950. doi: 10.1371/journal.pone.0285950 (PMC10198475; doi:10.1371/journal.pone.0285950)
Supplement: S4 Appendix — (DOCX) [file pone.0285950.s004.docx]

**S4 Appendix.** Percent excess deaths in Ukraine and other European countries, 2020.

(a) 30 European Countries

(b) Nearest European neighbors to Ukraine

Note: Estimates for Ukraine are based on Table 2. Estimates for other European countries are from Eurostat. S3 Appendix provides the list of countries used. Percentage excess deaths is calculated by (number of excess deaths/recorded deaths)X100. Excess death estimates produced by Eurostat are calculated from the average number of monthly deaths in each country during 2016-2019 and thus differed from our methodology. Max is the country with the highest percentage of excess deaths in any given month.
