# Supplementary material for: Impact of the COVID-19 pandemic on Ukrainian mortality, 2020–2021
Source: PLoS One. 2023 May 19;18(5):e0285950. doi: 10.1371/journal.pone.0285950 (PMC10198475; doi:10.1371/journal.pone.0285950)
Supplement: S5 Appendix — (DOCX) [file pone.0285950.s005.docx]

**S5 Appendix.** Recorded, basis, and excess deaths in 2020 by age group for June-December 2020; both sexes combined

| Age-Group (years) | Recorded  Deaths, number | Basis Deaths, number | Basis Deaths 95% Lower Bound, number | Basis Deaths 95% Upper Bound, number | Excess Deaths, number | Excess Deaths,^1^ percent | COVID-19 Coded Deaths, number | COVID-19 Coded Deaths,^1^ percent | Ratio of COVID-19 Coded Deaths to Excess Deaths |
| --- | --- | --- | --- | --- | --- | --- | --- | --- | --- |
| **2020 (June-December)** |  |  |  |  |  |  |  |  |  |
| 0-4 | 1,409 | 1,318 | 1,218 | 1,418 | 91 | 6.46 | 0 | 0.00 | 0.00 |
| 5-9 | 207 | 209 | 170 | 248 | -2 | -0.97 | 6 | 2.90 | -3.00 |
| 10-14 | 255 | 259 | 214 | 304 | -4 | -1.57 | 5 | 1.96 | -1.25 |
| 15-19 | 579 | 514 | 415 | 613 | 65 | 11.23 | 10 | 1.73 | 0.15 |
| 20-24 | 938 | 821 | 688 | 954 | 117 | 12.47 | 18 | 1.92 | 0.15 |
| 25-29 | 1,860 | 1,849 | 1,667 | 2,030 | 11 | 0.59 | 52 | 2.80 | 4.73 |
| 30-34 | 4,261 | 4,336 | 3,939 | 4,733 | -75 | -1.76 | 105 | 2.46 | -1.40 |
| 35-39 | 6,309 | 6,338 | 5,886 | 6,789 | -29 | -0.46 | 182 | 2.88 | -6.28 |
| 40-44 | 8,680 | 8,615 | 7,953 | 9,277 | 65 | 0.75 | 285 | 3.28 | 4.39 |
| 45-49 | 11,236 | 10,635 | 9,824 | 11,445 | 601 | 5.35 | 558 | 4.97 | 0.93 |
| 50-54 | 14,442 | 12,779 | 11,315 | 14,242 | 1,663 | 11.52 | 865 | 5.99 | 0.52 |
| 55-59 | 22,739 | 20,673 | 17,936 | 23,409 | 2,066 | 9.09 | 1,764 | 7.76 | 0.85 |
| 60-64 | 33,147 | 27,197 | 21,922 | 32,473 | 5,950 | 17.95 | 2,739 | 8.26 | 0.46 |
| 65-69 | 40,485 | 36,382 | 31,056 | 41,708 | 4,103 | 10.13 | 3,093 | 7.64 | 0.75 |
| 70-74 | 44,440 | 30,075 | 17,831 | 42,319 | 14,365 | 32.32 | 3,307 | 7.44 | 0.23 |
| 75-79 | 45,568 | 39,599 | 39,499 | 39,699 | 5,969 | 13.10 | 2,279 | 5.00 | 0.38 |
| 80-84 | 70,262 | 59,195 | 59,095 | 59,295 | 11,067 | 15.75 | 2,548 | 3.63 | 0.23 |
| 85+ | 65,376 | 55,927 | 55,827 | 56,027 | 9,449 | 14.45 | 1,163 | 1.78 | 0.12 |
| **Total** | **372,193** | **316,721** | **286,456** | **346,986** | **55,472** | **14.90** | **18,979** | **5.10** | **0.34** |

^1^ As a percent of recorded deaths
