# Supplementary material for: Impact of the COVID-19 pandemic on Ukrainian mortality, 2020–2021
Source: PLoS One. 2023 May 19;18(5):e0285950. doi: 10.1371/journal.pone.0285950 (PMC10198475; doi:10.1371/journal.pone.0285950)
Supplement: S6 Appendix — (DOCX) [file pone.0285950.s006.docx]

**S6 Appendix.** Recorded, basis, and excess deaths in 2021 by age group; both sexes combined

| Age-Group (years) | Recorded  Deaths, number | Basis Deaths, number | Basis Deaths 95% Lower Bound, number | Basis Deaths 95% Upper Bound, number | Excess Deaths, number | Excess Deaths,^1^ percent | COVID-19 Coded Deaths, number | COVID-19 Coded Deaths,^1^ percent | Ratio of COVID-19 Coded Deaths to Excess Deaths |
| --- | --- | --- | --- | --- | --- | --- | --- | --- | --- |
| **2021 (January -December)** |  |  |  |  |  |  |  |  |  |
| 0-4 | 2,346 | 2,335 | 1,993 | 2,680 | 11 | 0.47 | 27 | 1.15 | 2.6 |
| 5-9 | 328 | 357 | 222 | 491 | -29 | -8.84 | 5 | 1.52 | -0.2 |
| 10-14 | 500 | 405 | 251 | 560 | 95 | 19.00 | 12 | 2.40 | 0.1 |
| 15-19 | 987 | 813 | 472 | 1,152 | 174 | 17.63 | 25 | 2.53 | 0.1 |
| 20-24 | 1,564 | 1,386 | 928 | 1,843 | 178 | 11.38 | 79 | 5.05 | 0.4 |
| 25-29 | 2,868 | 3,118 | 2,494 | 3,736 | -250 | -8.72 | 168 | 5.86 | -0.7 |
| 30-34 | 6,988 | 7,298 | 5,937 | 8,658 | -310 | -4.44 | 383 | 5.48 | -1.2 |
| 35-39 | 11,424 | 10,759 | 9,210 | 12,307 | 665 | 5.82 | 782 | 6.85 | 1.2 |
| 40-44 | 15,667 | 14,647 | 12,379 | 16,917 | 1,020 | 6.51 | 1,283 | 8.19 | 1.3 |
| 45-49 | 21,176 | 18,282 | 15,503 | 21,062 | 2,894 | 13.67 | 2,130 | 10.06 | 0.7 |
| 50-54 | 27,018 | 22,309 | 17,290 | 27,327 | 4,709 | 17.43 | 3,310 | 12.25 | 0.7 |
| 55-59 | 40,536 | 36,061 | 26,678 | 45,443 | 4,475 | 11.04 | 5,815 | 14.35 | 1.3 |
| 60-64 | 63,388 | 47,723 | 29,636 | 65,810 | 15,665 | 24.71 | 10,366 | 16.35 | 0.7 |
| 65-69 | 78,225 | 63,416 | 45,152 | 81,672 | 14,809 | 18.93 | 13,881 | 17.74 | 0.9 |
| 70-74 | 95,629 | 53,304 | 11,328 | 95,286 | 42,325 | 44.26 | 16,512 | 17.27 | 0.4 |
| 75-79 | 78,888 | 72,575 | 72,301 | 72,987 | 6,313 | 8.00 | 11,176 | 14.17 | 1.8 |
| 80-84 | 138,318 | 104,658 | 105,707 | 106,393 | 33,660 | 24.34 | 13,612 | 9.84 | 0.4 |
| 85+ | 128,413 | 104,766 | 105,816 | 106,502 | 23,647 | 18.41 | 6,449 | 5.02 | 0.3 |
| **Total** | **714,263** | **564,214** | **463,299** | **670,827** | **150,049** | **21.01** | **86,015** | **12.04** | **0.6** |

^1^ As a percent of recorded deaths
