# Supplementary material for: Impact of the COVID-19 pandemic on Ukrainian mortality, 2020–2021
Source: PLoS One. 2023 May 19;18(5):e0285950. doi: 10.1371/journal.pone.0285950 (PMC10198475; doi:10.1371/journal.pone.0285950)
Supplement: S7 Appendix — (DOCX) [file pone.0285950.s007.docx]

**S7 Appendix.** Percent excess deaths by region and month, 2020

| Region | Total | June | July | Aug | Sept | Oct | Nov | Dec |
| --- | --- | --- | --- | --- | --- | --- | --- | --- |
| Vinnytsya | 21.1 | 10.7 | 11.8 | 3.4 | 19.6 | 18.6 | 37.5 | 33.1 |
| Volyn | 20.7 | 17.2 | 9.8 | 6.5 | 21.6 | 29.0 | 29.5 | 28.7 |
| Dnipropetrovsk | 21.9 | 7.4 | 4.4 | 1.9 | 16.6 | 33.9 | 43.4 | 40.3 |
| Donetsk | -1.1 | -19.2 | -18.9 | -18.1 | -6.2 | 12.9 | 5.1 | 15.8 |
| Zhytomyr | 21.0 | 15.2 | 3.8 | 0.9 | 15.4 | 25.8 | 41.8 | 34.0 |
| Zakarpattya | 23.3 | 20.3 | 31.4 | 14.3 | 22.2 | 18.3 | 34.0 | 22.0 |
| Zaporizhzhya | 16.7 | 2.8 | 3.7 | -5.2 | 9.5 | 14.2 | 39.2 | 49.1 |
| Ivano-Frankivsk | 26.9 | 13.6 | 19.0 | 20.9 | 30.5 | 19.2 | 40.0 | 30.7 |
| Kyiv | 23.5 | 14.4 | 6.2 | 2.3 | 21.2 | 23.0 | 33.1 | 51.5 |
| Kirovohrad | 21.4 | 11.2 | 16.1 | 4.4 | 13.4 | 11.7 | 38.9 | 49.4 |
| Luhansk | -1.9 | -24.4 | -18.9 | -11.5 | 9.6 | 9.3 | 3.7 | 16.6 |
| Lviv | 27.7 | 13.9 | 22.2 | 27.8 | 31.6 | 27.7 | 37.3 | 31.8 |
| Mykolayiv | 18.7 | 6.0 | 8.5 | -4.6 | 18.9 | 17.8 | 27.8 | 52.2 |
| Odesa | 17.7 | 3.2 | 8.5 | 3.3 | 20.5 | 13.7 | 27.3 | 44.1 |
| Poltava | 21.1 | 12.1 | 6.2 | 2.3 | 7.7 | 19.4 | 47.5 | 46.4 |
| Rivne | 19.9 | 11.4 | 8.2 | 17.0 | 12.1 | 15.9 | 36.6 | 34.9 |
| Sumy | 25.1 | 16.4 | 16.0 | 1.8 | 21.0 | 33.6 | 47.4 | 36.4 |
| Ternopil | 21.3 | 10.7 | 3.7 | 5.3 | 39.0 | 18.5 | 39.8 | 31.2 |
| Kharkiv | 21.6 | 8.7 | 7.9 | 3.2 | 24.2 | 37.0 | 33.2 | 33.6 |
| Kherson | 19.8 | 8.2 | 9.8 | -2.5 | 12.4 | 9.7 | 28.8 | 65.3 |
| Khmelnytskiy | 20.5 | 10.5 | -2.7 | 7.0 | 20.5 | 29.0 | 43.5 | 31.0 |
| Cherkasy | 16.9 | 8.7 | 3.8 | -8.0 | 9.2 | 13.3 | 33.5 | 51.5 |
| Chernivtsi | 28.0 | 18.6 | 16.4 | 17.9 | 34.1 | 28.9 | 32.7 | 45.1 |
| Chernihiv | 20.6 | 7.9 | 9.8 | -4.7 | 18.5 | 23.2 | 44.9 | 39.3 |
| Kyiv city | 19.1 | 11.3 | -3.4 | -2.7 | 13.4 | 21.1 | 45.3 | 43.3 |

*Note*: Excess deaths as a percent of recorded deaths.
