# Supplementary material for: Impact of the COVID-19 pandemic on Ukrainian mortality, 2020–2021
Source: PLoS One. 2023 May 19;18(5):e0285950. doi: 10.1371/journal.pone.0285950 (PMC10198475; doi:10.1371/journal.pone.0285950)
Supplement: S8 Appendix — (DOCX) [file pone.0285950.s008.docx]

**S8 Appendix.** Percent excess deaths by region and month, 2021

| Region | Total | Jan | Feb | March | April | May | June | July | Aug | Sept | Oct | Nov | Dec |
| --- | --- | --- | --- | --- | --- | --- | --- | --- | --- | --- | --- | --- | --- |
| Vinnytsya | 21.5 | -3.2 | 12.5 | 45.8 | 43.9 | 8.7 | 10.9 | 5.6 | -0.9 | 13.1 | 21.0 | 59.5 | 39.8 |
| Volyn | 19.4 | -11.5 | 5.1 | 13.3 | 28.3 | 16.0 | 18.1 | 6.8 | -9.1 | 18.3 | 34.6 | 83.4 | 33.8 |
| Dnipropetrovsk | 32.8 | 6.2 | 9.0 | 26.9 | 53.2 | 27.2 | 17.5 | 8.7 | 9.7 | 16.0 | 81.6 | 107.6 | 28.9 |
| Donetsk | 12.1 | -5.0 | -7.0 | 3.5 | 16.0 | 1.2 | -1.3 | -3.3 | -11.0 | 2.3 | 50.8 | 77.6 | 16.8 |
| Zhytomyr | 23.8 | 1.3 | 10.8 | 37.9 | 47.6 | 7.4 | 17.1 | 2.5 | -0.2 | 23.1 | 41.9 | 65.4 | 29.8 |
| Zakarpattya | 23.6 | -4.2 | 22.4 | 69.7 | 29.5 | 4.2 | 20.4 | 4.8 | -2.9 | 17.9 | 16.3 | 55.8 | 45.8 |
| Zaporizhzhya | 31.6 | 10.6 | 3.6 | 18.0 | 38.4 | 19.1 | 5.0 | 5.9 | 2.3 | 21.8 | 90.8 | 131.5 | 31.8 |
| Ivano-Frankivsk | 21.4 | -2.5 | 26.2 | 58.4 | 18.1 | 10.4 | 7.9 | 6.6 | 0.3 | 18.9 | 24.9 | 56.4 | 28.0 |
| Kyiv | 28.6 | 9.0 | 11.5 | 38.1 | 62.6 | 12.1 | 20.2 | 6.3 | 6.9 | 21.9 | 38.5 | 79.1 | 35.4 |
| Kirovohrad | 30.6 | 8.6 | 5.5 | 22.8 | 52.1 | 30.1 | 13.9 | 7.3 | 7.0 | 10.9 | 28.8 | 97.9 | 80.1 |
| Luhansk | 9.4 | -0.8 | 0.8 | 7.4 | 10.2 | -1.5 | 1.5 | -1.3 | -9.9 | 16.2 | 33.4 | 47.4 | 8.6 |
| Lviv | 24.7 | 2.0 | 7.3 | 27.8 | 41.0 | 13.3 | 16.6 | 2.9 | 3.6 | 27.6 | 49.9 | 75.3 | 30.2 |
| Mykolayiv | 32.2 | 10.6 | 14.1 | 30.8 | 65.7 | 17.0 | 12.9 | 9.5 | 1.5 | 11.1 | 53.5 | 113.7 | 42.8 |
| Odesa | 33.3 | 6.9 | 10.6 | 31.4 | 61.6 | 15.2 | 15.7 | 13.5 | 4.8 | 23.3 | 81.9 | 104.5 | 30.5 |
| Poltava | 26.2 | 4.3 | 8.6 | 20.1 | 36.2 | 18.6 | 14.4 | 5.5 | 3.6 | 17.7 | 39.2 | 92.6 | 51.3 |
| Rivne | 22.6 | 2.0 | 9.1 | 16.6 | 31.1 | 29.1 | 13.2 | 1.5 | 3.9 | 16.7 | 40.9 | 80.7 | 24.7 |
| Sumy | 28.8 | 6.4 | 7.5 | 20.9 | 47.6 | 16.0 | 20.2 | 9.1 | -0.6 | 23.4 | 60.2 | 105.8 | 31.6 |
| Ternopil | 18.0 | -5.5 | 8.1 | 26.0 | 38.7 | 11.3 | 10.9 | -0.8 | -9.0 | 15.3 | 35.3 | 57.5 | 27.5 |
| Kharkiv | 38.5 | 10.1 | 14.5 | 28.1 | 61.7 | 23.1 | 16.4 | 9.8 | 7.8 | 25.3 | 103.4 | 128.3 | 31.1 |
| Kherson | 31.3 | 10.3 | 9.0 | 14.2 | 28.6 | 17.5 | 8.7 | 10.6 | 10.0 | 28.6 | 97.5 | 113.7 | 28.5 |
| Khmelnytskiy | 26.3 | 6.3 | 8.3 | 33.7 | 64.9 | 16.8 | 9.9 | 0.6 | 3.1 | 16.5 | 37.0 | 76.3 | 38.7 |
| Cherkasy | 19.5 | 2.6 | 8.6 | 19.7 | 31.8 | 12.7 | 15.4 | 4.1 | 1.6 | 8.2 | 31.6 | 70.4 | 26.9 |
| Chernivtsi | 36.5 | -0.8 | 33.3 | 65.0 | 50.5 | 17.7 | 17.5 | 6.3 | 13.0 | 47.7 | 75.8 | 77.0 | 35.3 |
| Chernihiv | 20.0 | 4.4 | 6.1 | 15.9 | 39.8 | 16.2 | 15.0 | 3.7 | 0.9 | 16.2 | 26.2 | 63.5 | 31.8 |
| Kyiv city | 33.4 | 16.2 | 20.7 | 51.8 | 68.0 | 28.1 | 12.6 | 9.5 | 2.9 | 16.3 | 53.1 | 86.4 | 29.1 |

*Note*: Excess deaths as a percent of recorded deaths.
